# A90 WHEN DO TRAINEES ACHIEVE COMPETENCY IN PERFORMING ENDOSCOPIC SUBMUCOSAL DISSECTION: A SYSTEMATIC REVIEW

**DOI:** 10.1093/jcag/gwab049.089

**Published:** 2022-02-21

**Authors:** R Kaviani, M Tomaszewski, N C Shahidi

**Affiliations:** Internal Medicine, The University of British Columbia, Vancouver, BC, Canada

## Abstract

**Background:**

Endoscopic submucosal dissection (ESD) is an established organ sparing curative endoscopic resection technique for the management of pre-malignant and superficially invasive malignant lesions of the gastrointestinal tract. However, little is understood of its learning curve with suggested competency measures including en bloc resection, R0 resection, adverse events and resection speed.

**Aims:**

We aimed to perform a systematic review on when competency is achieved in ESD.

**Methods:**

Two authors independently searched MEDLINE and EMBASE (1946 to Aug 2021) for full-text original citations including grey literature assessing the ESD learning curve. A learning curve was defined as an assessment of competency as a function of increasing trainee experience. Thresholds for competency were defined as en bloc resection ≥ 80%, R0 resection ≥ 80%, perforation rate ≤ 5% and resection speed ≤ 6.67min/cm^2^.

**Results:**

Forty-three studies (1 esophageal, 11 gastric, 27 colorectum, 4 multiple sites) with 157 trainees and 8780 ESD procedures were included. Baseline experience in esophagogastroduodenoscopy, colonoscopy, endoscopic mucosal resection and ESD were 800–10,000, 100–10,000, 4–700 and 0–300 procedures, respectively. 16 studies used animal model training prior to assessing the ESD learning curve.

En bloc resection, R0 resection, perforation rate and resection speed were used as markers of competency in 33, 29, 28 and 21 studies, respectively. When pooling evaluations where competency was achieved, it was reached in the esophagus, stomach, colorectum and for multiple sites between <10, <10–150, <10–301, and <20–300 procedures, respectively (Table 1). However, competency in R0 resection, perforation rate, and resection speed was not uniformly achieved within 12 studies.

**Conclusions:**

Competency in ESD can be achieved in <10 - 399 procedures, with variability dependent on organ site and baseline level of training. With the widespread adoption of ESD, standardization of training and the assessment of competency are needed.

**Funding Agencies:**

None

**Table:** Table 1. ESD competency outcomes by site and number of procedures

Competency **Achieved**	En bloc **≥ 80%**	R0 **≥ 80 %**	Perforation Rate **≤ 5%**	Resection Speed **≤ 6.67min/cm**^**2**^
Esophagus	<10	<10	<10	-
Stomach	<10 - 38	<12 - 100	<10 - 150	-
Colorectum	<12 - 180	<16 - 301	<10 - 250	25 - 240
Multiple Sites	<20 - 200	21 - 300	25 - 36	300

